# Evaluation of the Functional Movement Screen (FMS) in Identifying Active Females Who are Prone to Injury. A Systematic Review

**DOI:** 10.1186/s40798-021-00380-0

**Published:** 2021-11-22

**Authors:** Mojtaba Asgari, Shahab Alizadeh, Anna Sendt, Thomas Jaitner

**Affiliations:** 1 Institute for Sport and Sports Science, TU Dortmund University, Otto-Hahn- Straße 3, 44227 Dortmund, Germany; 2 School of Human Kinetics and Recreation, Memorial University of Newfoundland, St. John’s, Newfoundland and Labrador Canada

**Keywords:** Sport injuries, Injury prediction, Functional movement screening, FMS, Female athletes

## Abstract

**Background:**

The validity of the Functional Movement Screen (FMS) in identifying active females who are predisposed to injury has not been specifically reviewed. This study aims to synthesize the literature on the ability of the FMS to identify at-risk active females.

**Methods:**

Six online databases, including PubMed, Medline, Web of Science, Science Direct, SPORTDiscus and Google Scholar, were searched for the period of April 2006 to September 2021. Out of the 61 potential references, 17 were reviewed in detail with respect to the inclusion criteria; ten were ultimately included. The risk of bias, applicability and level of the studies were then identified using the QUADAS-2 and a checklist for assessing methodological quality. The following data were obtained from the included studies: year of publication, title, study type, participants’ demographic, sample size, FMS cutoff point, injury definition, statistical analyses used, FMS results and study level.

**Results:**

Generally, the quality of eight studies was poor to moderate due to both small sample sizes and short follow-up periods. Except for a study on military members, all studies were carried out on team sports players. The overall bias of the studies was low, but there was an unclear amount of bias for participant selection. Two studies reported no predictive validity for the FMS, while three defended its predictive validity; the rest partially supported the FMS as a valid diagnostic tool. The reliability of the recommended cutoff point was confirmed, though cutoffs higher than 14 were significantly associated with the predictive ability of the FMS.

**Conclusion:**

Although the FMS is reliable for clinical practice, and the current literature shows promise regarding the predictive ability of the FMS among active females, concerns remain regarding its validity in identifying at-risk females. Given the lack of clarity in the literature on the use of the FMS in females, further well-organized studies with larger sample sizes and longer monitoring periods are highly recommended. The sensitivity and specificity of the recommended cutoff of ≤ 14 has considerably decreased , and higher cutoff values should be applied to increase the FMS predictive ability.

*Level of evidence* The level of evidence was determined to be 2b.

## Key Points

- The Functional Movement Screen (FMS) was identified to be the most popular field-based injury screening tool for identifying at-risk athletes
- It could not identify male athletes who are prone to injury
- For female athletes, concerns remain regarding the FMS predictive validity due to the poor and contradictory nature of the available literature.
- Further well-established studies involving only females are needed to eliminate sex bias in the FMS literature
- Cutoff values higher than 14 increase the sensitivity of the FMS in identifying at-risk females

## Introduction

The 2012 London Olympics was labeled the ‘‘Year of the Woman’’, since each delegate sent at least one female athlete to compete in the games, with women accounting for nearly 45% of the entire population of athletes [1]. These statistics highlight the increase in the number of females participating in competitive and recreational sports. Despite the numerous advantages of being physically active, taking part in either competitive or recreational sports is accompanied by an increase in injury risk [2]. Extensive studies, mainly in the form of sophisticated biomechanical analyses, have determined the injury risk factors [3–10], providing great potential for injury prevention. These laboratory-based measures provide precise quantification of the presumed risk factors, but they entail costly equipment and large amounts of time, so performing them on large scales is impractical. Therefore, identifying at-risk athletes via demographics has become an area of interest.

In a scientific endeavor, a series of field-based screening tools such as the Y Balance Test (YBT), Star Excursion Balance Test, Landing Error Scoring System (LESS) and Functional Movement Screen (FMS) were developed as more user-friendly alternatives to the laboratory measures [11–15]. Despite the compromises made for precision that these tools entail, they are inexpensive, easy to operate and efficient to use in large-scale settings and require less effort than the laboratory investigations such as isokinetic dynamometry, 3D motion capturing, and EMG. They have been shown to be practical in identifying a lack of neuromuscular control/imbalance [16, 17] as well as poor core stability and strength [18], which are known as risk factors for musculoskeletal injuries [11, 19–22]. Given this, they serve as diagnostic tools, particularly in team sports, so that injury prevention programs can be tailored based on the results [11, 12].

The FMS, among other tools, has demonstrated good to excellent interrater and intra rater reliability in discerning deficits in movement behavior and motor function that are assumed to be related to injury risk [23, 24] and has thus received much attention in recent years. It targets seven fundamental movement patterns involving balance, mobility and stability that reflect fundamental proprioception and kinesthetic awareness principles [25]. Each component involves a specific movement pattern that challenges the body’s kinetic function as a linked system [26] and is designed to provide a qualitative assessment of locomotion and stability [25, 27]. The quality of the components is rated on a zero-to-three scale, zero indicating pain when performing the movement, one indicating poor performance and inability to complete the task, two indicating the use of compensatory movement patterns, and three indicating excellent performance [25]. A composite score is calculated as the sum of the ratings for all seven components. The Composite scores less than 14 are associated to a high risk of injury, other cutoffs have also been applied [28, 29].

Although the results have been contradictory, many studies have evaluated the ability of the FMS to replace costly laboratory tools [30–32]. Kolodziej and Jaitner (2018), for example, demonstrated that the composite FMS score is a valid indicator of injury risk in amateur male soccer players [32], whereas Bardenett et al. (2015) reported that it is not a valid predictor of injury in male and female high school athletes [33]. More recently, the validity of the FMS in identifying individuals predisposed to injury has been seriously challenged in a number of reviews [24, 34, 35]. Nevertheless, the preceding reviews examined either only males or mixed samples and, to the knowledge of the authors, no systematic review considering only females has been performed to date. This is a prominent issue given that the musculoskeletal as well as physiological features of each sex affect not only intrinsic events but also movement patterns/behavior and the mechanism, type and overall risk of injury. Finally, but importantly, sex has been found to be a significant variable in FMS studies [36], and Gnacinski et al. (2016) stated that a sex bias exists in the FMS literature, i.e., the FMS sum score is not equally meaningful for males and females [38]. The present study, therefore, aims to synthesize the available FMS-related literature in the form of a systematic review to address whether the FMS can be used as a diagnostic tool for identifying active females who are at higher risk of injury.

## Methods

### Search Protocol and Registration

This study was performed in accordance with the Preferred Reporting Items for Systematic Reviews and Meta-Analyses (PRISMA) guidelines and was not preregistered [37, 38]. Two blinded members of the research group (MA and SA) independently and systematically searched six online databases (PubMed, Medline, Web of Science, Science Direct, SPORTDiscus and Google Scholar) for the period from 2006 (year of introduction of the FMS) to September 2021. Additionally, a manual search of the references was carried out to further identify papers, minimizing the probability of missing related references.

### Search Procedure, Eligibility Criteria and Study Selection

Three main predefined keywords comprised of “Functional Movement Screen”, “musculoskeletal screening” and “FMS” were used in conjunction with the keywords females, girls, women, athletes, healthy individuals, injury, prediction, pre-assessment, pre-participate, functional assessment and movement quality assessment to identify potentially relevant studies. Excluding systematic reviews, meta-analyses and duplicates, all studies examining the FMS were considered in the primary assessment. Initially, titles were checked, and relevant papers were further considered for abstract assessment. The abstracts were then scrutinized to identify female-only studies using Endnote software (Clarivate Analytics, Boston, MA, USA) identical to the method proposed by Bramer et al. [39]. Finally, references that met the following criteria were considered for full-text review (details are presented in figure one): (1) investigated the ability of the FMS in injury prediction, (2) had female or mixed samples with results presented according to sex, (3) had a prospective design and (4) published in English. There were no limits imposed on vocation, level of physical activity or sport or the characteristics of the participant sample.

### Data Collection and the Level of the Studies

**Fig. 1:**
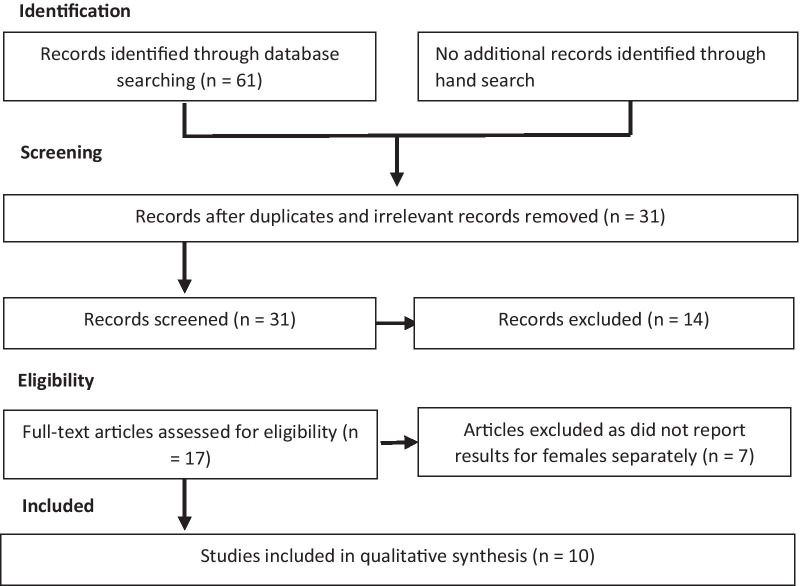
PRISMA flow chart

Out of the 31 articles that met the inclusion criteria, 17 full texts were screened in detail for eligibility, of which 10 qualified for the systematic review [28, 31, 40–47]. The main reason for exclusion was recruiting a mixed sample without reporting the results for females separately. Two independent coauthors (MA and SH) reviewed the articles in detail and compiled the extracted data in an Excel spreadsheet (Microsoft, Redmond, WA, USA). These data included the author’s name, year of publication, title, type of study, sex, sample size, cutoff, injury definition, statistical analyses used and whether the results showed a significant difference between the FMS scores of the injured and uninjured participants. The level of the studies was also re/evaluated by adopting a checklist for assessing methodological quality that was produced by Downs et al. (1998) with the study classification form of the Center for Evidence-Based Medicine, Oxford-WILEY, online publication [48] (Fig. 1).

### Risk of Bias and Validity of Individual Studies

According to the guidelines of the QUADAS-2, a highly recommended tool for accuracy assessment in systematic reviews [49], the applicability as well as the risk of bias of the eligible references were double-checked by two coauthors (AS and SA). They reviewed and scored the papers independently. When conflicts occurred, the relevant article was re-scored by the lead researcher. Comparing the scores revealed that except for an unclear amount of bias in subject selection in some studies, applicability concerns as well as risk of bias for all domains were low; details are included in Table 1.

**Table 1:** Risk of bias and applicability

Study	Risk of bias				Applicability concerns		
	Patient selection	Index test	Reference standard	Flow and timing	Patient selection	Index test	Reference standard
Armstrong et al. 2018 [40]	?	✓	✓	✓	✓	✓	✓
Walbright et al. 2017 [42]	?	✓	✓	✓	✓	✓	✓
Clay et al. 2016 [28]	?	✓	✓	✓	✓	✓	✓
Knapik et al. 2015 [43]	✓	✓	✓	✓	✓	✓	✓
Landis et al. 2018 [41]	✓	✓	✓	✓	✓	✓	✓
Pfeifer et al. 2019 [47]	?	✓	✓	✓	✓	✓	✓
Gonzalez et al. 2018 [44]	?	✓	✓	✓	✓	✓	✓
Kodesh et al. 2015 [46]	✓	✓	?	?	✓	✓	✓
Chorba et al.2010 [31]	?	✓	✓	✓	✓	✓	✓
Šiupšinskas et al. 2019 [45]	✓	✓	✓	✓	✓	✓	✓

## Results

Table 2 presents a summary of the studies including the author, year of publication, participant information, study design, statistical methods used, findings and level of the study. The studies all applied different aspects of the same definition of injury in accordance with the consensus statement on injury definitions and data collection procedures: an incident that prevents the participant from practicing for at least one day [50]. Overall, the quality of eight included studies was poor to moderate due to both small sample sizes and short follow-up periods, and the remaining studies were of moderate to high quality.

**Table 2:** Summary of the included articles

Study	Participants	Study design	Statistical methods	Results	Level of evidence	Cut-off
Chorba et al. 2010 [31]	38 NCAA Div. II female collegiate football, volleyball and basketball players	Prospective cohort	A Fisher's exact test with a one-tailed p value of < 0.05 Correlation and regression analyses	Predisposed females can be identified by using a functional movement screening tool	2b	≤ 14
Kodesh et al. 2015 [46]	158 female soldiers of Combat Fitness Instructor Course of the Israel Defense Forces	Prospective cohort	Fisher Exact Test to evaluate association of the FMS scores and incidence of injuries in high/low risk groups	FMS is defective in predicting injury predisposed female soldiers	2b	≤ 14
Knapik et al. 2015 [43]	275 females of Coast Guard cadets	Prospective cohort	Chi-square statistics risk ratio (RR) and 95% confidence interval (95% CI) 2 × 2 contingency tables (assessing Sensitivity and specificity) The Youden’s Index (to determine the FMS total score cut point that optimized sensitivity and specificity)	Functional movement screening demonstrated moderate prognostic accuracy for determining injury risk among female Coast Guard cadets	2b	≤ 15
Clay et al. 2016 [28]	37 Division. I female collegiate rowers and coxswains	Prospective cohort	1.Chi-square to determine significant associations between FMS group and history of injury 2.Fisher’s Exact tests to see if any cells in the 2 × 2 contingency tables were less than 10	No statistically significant evidence for prediction of time loss injury was observed	2b	≤ 14
Walbright et al. 2017 [42]	35 female collegiate volleyball and basketball players	Prospective cohort	1.ROC analyses 2.Assessment of the area under curve 3.Two sided Z test to determine differences	No adequate validity to predict lower quarter injury risk was reported	2b	≤ 14
Armstrong et al. 2018 [40]	64 female university rugby union players	Prospective cohort	1.ROC analyses 2.Linear regression, multiple linear regression and stepwise multiple hierarchical linear regression analyses	Individual components of the FMS are a better predictor of injury than FMS composite score	2b	≤ 14 to ≤ 16
Landis et al. 2018 [41]	187 collegiate female football, volleyball and basketball players	Prospective cohort	1. Univariate analyses 2. Independent samples t-test to compare mean data of the groups	The FMS can be used to identify athletes at an increased risk of sustaining a non-contact ACL or Lower Extremity injury	3a	≤ 14
Gonzalez et al. 2018 [44]	31 National Collegiate Athletic Association Division I, female, open-weight rowers	Prospective cohort	1. Chi-square statistic to determine association of history of LBP and experience LBP during the current season 2. independent-samples t tests 3. multiple regression analysis 4. Receiver operating characteristic (ROC)	The FMS is not recommended for widespread screening of female rowers because the risk ratio was relatively small and had a wide 95% confidence interval	2b	11.5to14.5
Šiupšinskas et al. 2019 [45]	169 elite female basketball players	Prospective cohort	1.Student’s t-test 2.The Mann–Whitney U-test	A combination of functional tests can be used for injury risk evaluation in female basketball players	2b	< 15
Pfeifer et al. 2019 [47]	73 female Sport local schools inc. Football, soccer, volleyball, lacrosse	Prospective cohort study	1.power analysis 2.Independent t-tests 3.logistic regression	A composite FMS score of < 14 or < 15 was associated with an increased risk of sustaining injury (OR = 2.99)	1b	< 14 or < 15

Chorba et al. (2010) implemented the first female-only study on the FMS. Thirty-eight female collegiate athletes (mean age 19.24 ± 1.20 years) from the National Collegiate Athlete Association (NCAA) participating in soccer, volleyball and basketball took part in the study. A preseason FMS assessment, followed by monitoring during the subsequent competitive season, was carried out. The mean FMS scores for individuals who sustained injuries and those who did not were 13.9 ± 2.12 and 14.7 ± 1.29, respectively. The 81.82% of participants who scored ≤ 13 and 48.28% of those who scored ≤ 15 sustained an injury, demonstrating a correlation between the FMS composite score and the risk of injury. A strong correlation existed between injury and FMS score (*r* = 0.761, *P* = 0.021), and those with an FMS composite score of ≤ 14 were found to be significantly more likely to sustain an injury (*P* = 0.0496). Interestingly, participants with a previous history of injury had lower FMS scores and were more susceptible to recurrent injuries. However, statistical analysis revealed a predictive relationship between the FMS score and the risk of injury only for participants without a history of ACL injury whereas when expanded to all participants, the linear regression failed to reach statistical significance. Hence, this study partially confirmed the predictive value of the FMS in female collegiate athletes [31].

Kodesh et al. (2015) evaluated the FMS as a predictive tool in 185 soldiers from the Israel Defense Forces (age range: 18.1–20.2). The injury risk of the participants was examined using the FMS before a three-month course in combat fitness including endurance and resistance training plus regular military training. A total of 147 injuries occurred in 97 soldiers, with 80% of injuries occurring in the lower extremity (LE), and most injuries being caused by overuse (84%). The mean FMS score among all participants was 16, with the highest score occurring most frequently for shoulder mobility (score 3), whereas 51.35% of the injured group and 30.5% of the non-injured group scored zero in one or more movement patterns. The recommended FMS cutoff only predicted 42% of those who reported an injury, and it correctly identified 63% of those who did not report an injury, indicating poor overall sensitivity and specificity. With a cutoff of 14, no significant difference was observed between the injured (range 7–20, interquartile range; 12.75–18.0) and non-injured (range 2–21, interquartile range; 13.25–17.0) groups (*p* = 0.70), and the FMS did not predict injuries in females during the military training [46].

Knapik et al. (2015) illustrated the ability of the FMS to identify injury occurrence in 275 Coast Guard cadets. After a preseason assessment using the FMS, participants were monitored over an 8-week Summer Warfare Annual Basic (SWAB) intensive training. The cutoff that maximized specificity and sensitivity was determined from the Youden’s index; composite scores ≤ 15 were associated with higher injury risk than scores ≥ 15. However, the Youden’s Index indicated that the optimal FMS cutoff for women was ≤ 14 (60% sensitivity, 61% specificity), and with this cutoff, the injury risk among women was greater for those with lower FMS composite scores. As a result, FMS composite scores < 15 were associated with a higher injury risk than scores > 15, suggesting moderate prognostic accuracy of the FMS for determining injury risk among female Coast Guard cadets. Based on this investigation, it is argued that predicting injury risk using the FMS test has limited promise [43].

A clinical study by Clay et al. (2016) investigated whether the FMS score could serve as a predictor of the incidence of (1) time-loss injuries and (2) low back pain (LBP) in rowers and coxswains. In the off-season, the participants completed the Oswestry LBP as well as the first part of a rowing-specific questionnaire followed by the FMS test. Injury reports and patient complaints regarding LBP during the subsequent rowing season were collected and compared to the FMS scores. Results revealed that the high risk group was significantly more likely to experience LBP during the season (*p* = 0.036) and had a 58% greater mean in years of rowing experience (*p* = 0.008) than individuals in the low risk group. Additionally, those with a history of LBP were six times more likely to experience LBP during the season (*p* = 0.027). Although the high-risk group had a 30% greater occurrence of injury, no statistically significant trend in the injury rate by group was observed. The authors concluded that the FMS is not a sufficient predictor of reported time-loss injuries in female rowers but that it did predict the incidence of LBP [28].

Walbright et al. (2017) conducted a clinical study to examine the validity of three common functional screening tools including the YBT, FMS and single-leg hop test (SLHT) in predicting LE injuries in female collegiate volleyball and basketball players. During a preseason assessment, the tests were utilized, and further injuries incurred during the subsequent 33-week course of training and competition were recorded. The results revealed a high true positive rate for the FMS components: squat, hurdle step, inline lunge and rotary stability; conversely, a high true negative rate was observed for the straight leg raise. However, the positive and negative likelihood ratios both showed a lack of predictive value for any FMS component in predicting the incidence of time-loss injuries. Additionally, the mean FMS score for all participants was 14.9 ± 1.7, with the mean for those with lower quarter injuries (LQIs) being 14.6 ± 1.6 and that for those without LQIs being 15.4 ± 1.9, depicting a slightly better but not significantly higher score than that of the injured participants. As result, a non-significant relationship between LQIs with time loss and the YBT, FMS (composite/component) and SLHT scores was reported. The screening tests within this study implied a lack of validity in the prediction of LE injuries among female collegiate basketball and handball players [42].

Gonzalez et al. (2018) undertook a prospective cohort study to determine whether the FMS could identify open-weight female rowers at greater risk of LBP, defined as LBP (1) occurring as a result of rowing training, (2) resulting in at least 1 day of missed practice or competition, and (3) resulting from a diagnosed injury of any lumbar spine muscle, joint, tendon, bone, nerve or disk or from nonspecific LBP. In total, 31 female rowers took part in the study, of which 18 (age = 19.9 6 ± 1.4 years) experienced an episode of LBP; there was not a considerable difference in the FMS composite score between the injured and non-injured groups with the given cutoff, while using a cutoff of 16 led to a significant difference between the groups. Rowers with scores less than 16 had a 1.4 times higher chance of experiencing an episode of LBP, in addition to a shorter plank test time. Although the FMS did predict LBP in female rowers, the relative risk was low (1.4), and thus, the results cannot be generalized to all female rowers. The FMS, therefore, was not recommended for widespread screening of female rowers because the risk ratio was relatively small and had a wide 95% confidence interval [44].

An epidemiological observational study carried out by Landis et al. (2018) documented that female athletes (mean age 19.5 ± 1.21 years) playing collegiate football, volleyball and basketball who scored 14 or less on a preseason evaluation had a significantly greater chance of sustaining a noncontact LE injury (*t* = 1.98, *p* = 0.049, 95% CI = 0.01, 2.69). Accordingly, compared to the non-injured participants (15.35 ± 2.58), the injured participants had a significantly lower mean composite FMS score (14 ± 3.46). Interestingly, those who had already suffered an ACL injury had a lower mean average FMS composite score (12 ± 4.83) than participants without a history of ACL injury (15.3 ± 2.61, *t* = 2.452, *p* = 0.015, 95% CI = 0.644, 5.948), indicating the necessity of considering a previous injury as the main risk factor for recurrent injuries. The injured participants showed diminished movement quality for the following FMS components: lunges, straight leg raise, push-ups, trunk rotation stability and deep squats. The authors concluded that the FMS composite score can be used to identify female athletes who are prone to noncontact LE and ACL injuries [41].

Armstrong et al. (2018) compared the FMS composite and individual component scores as a predictor of total days injured (TDI) and reported excellent intra rater reliability (ICC) (0.99, CI: 0.97–0.99) for the FMS composite score. The participants were examined during a preseason FMS test and monitored for injuries over the course of a rugby season. Composite score values from 11.5 to 14.5 served as cutoffs for predicting injury. The results showed that the injury rates for females were 5.80 and 55.56 per 1000 h of training and competition, respectively. Small Cohen’s d effect sizes for the FMS composite score as a predictor of TDI were reported for the combined sample (0.19), males (0.20) and females (0.27). Findings revealed that the FMS could be used to identify university-level female rugby players (age 20.39 ± 1.91 years) at risk of injury, while the individual components of the FMS were better predictors than the FMS composite score. It was implicitly stipulated that the FMS composite score is a weak predictor of TDI [40].

Šiupšinskas et al. (2019) performed a set of preseason screening tests including the YBT, FMS and LESS on 169 female basketball players and tracked the occurrence of LE injuries over three seasons (2013–2016). A total of 92 LE injuries were recorded, of which 40.2% occurred in the knee, with the highest frequency being for ACL, MCL and LCL injuries (*n* = 22, 21.7%), and 38% occurred in the ankle with the highest frequency being for acute ligament injuries (*n* = 14, 15.2%) and chronic ankle ligament tendinopathy (*n* = 13, 14.1%). Lower FMS composite scores in female basketball athletes were associated with LE injuries, and athletes from the injured group scored 1.3 points lower on their total FMS score than non-injured players (14.1 vs 15.4, *p* = 0.0001). However, this study suggests combination of functional tests can be used for pre-participation screening [45].

Pfeifer et al. (2019) studied 73 female football, volleyball and lacrosse athletes (aged 11–18 years, mean 16.01 + 1.35) to determine the ability of the FMS to identify those predisposed to injury. The participants performed the FMS test prior to each competitive season and were monitored for injuries during the following competition season. Statistical analyses showed that females had significantly higher mean FMS composite scores than males (*f* = 14.40; *m* = 12.62; *p* < 0.001) and better component scores for the hurdle step, shoulder mobility, active straight leg raise and rotary stability components. The authors indicated that a composite FMS score of < 14 or < 15 was associated with an increased risk of injury (OR = 2.99) and concluded FMS alone may not adequate for the prediction of injury, and that the screen should be supplemented with other measures of sport readiness [47].

## Discussion

The goal of this systematic review was to clarify the validity of the FMS in identifying active females who are susceptible to injury. Despite numerous studies on different features of the FMS, scrutinizing the available literature revealed that females have not been adequately studied with the FMS; the literature is sparse, poor and contradictory. Thus, further well-established studies involving only females are needed to eliminate sex bias in the FMS literature.

Briefly, out of 61 original references found in our search, 17 full texts were screened in detail, ten of which were ultimately included in the study. Overall, three studies [43–45] indicated that using a cutoff higher than 14 would noticeably increase the sensitivity of the FMS, demonstrating that utilizing higher cutoff points than the recommended value of ≥ 14 would significantly increase the predictive validity of the FMS. These data, alongside the study of Stacy et al. (2016), support that practitioners should use different FMS cutoff values when evaluating female populations [51]. Moreover, Kodesh et al. proved that the recommended FMS cutoff point only predicted 42% of injured and 63% of non-injured athletes, illustrating poor overall sensitivity and specificity. In a critical review, Bahr (2016) commented that there needs to be further clarity of the application of cutoff values for high and low risk groups and at what level the cutoff should be set. He considered this issue as a second step of validating the screening tests and suggested that if the intervention is easy and has no side effects (both are advantages of the FMS) a cutoff with more sensitivity is more reasonable than a conservative cutoff (high specificity) [52]. Therefore, a consistency exists between the current literature and Bahr’s statement regarding applying cutoff values higher than 14, and this must be considered in future studies on female populations.

Moreover, the studies of Knapik et al. (2015) and Pfeifer et al. (2019) demonstrated that applying the FMS composite score alone would be insufficient for screening active females, and a more specialized screening approach may be more practical for accurately estimating the risk of injury [43, 47]. These data reaffirm outcomes of the previous studies [53, 54] indicating that using the FMS in conjunction with evaluating other physical readiness parameters such as power and endurance may predict at-risk individuals more accurately. Further well-organized female only studies should also document this assumption. Additionally, Armstrong et al. (2018) emphasized that the individual components of the FMS are better predictors than the FMS composite scores [40], whereas Walbright et al. (2017) showed a lack of predictive value for any FMS component [42]. Given the lack of clarity in the literature, future studies should tackle this challenge while considering the specific characteristics of the population being assessed. Along this line, even in male populations, only a few previous studies have compared individual components of the FMS to injury risk [54–56], where the results indicate that the individual components are stronger predictors of injury than the FMS composite score.

In general, the available literature on the use of the FMS in active females falls into three main categories. First, there were studies that pointed out the validity of the FMS in discerning female athletes at higher risk of injury, including Armstrong et al. (2018), Landis et al. (2018) and Šiupšinskas et al. (2019) albeit the latter recommended a combination of functional tests can be useful for screening [40, 41, 45]. The participants of these studies included football, basketball, volleyball, rugby and lacrosse players, indicating that the FMS composite score might be a valid predictor of injury among active females participating in overhead and contact sports. This hypothesis, however, is in contrast to the results of Walbright et al. (2017) who illustrated a lack of validity for the FMS as an LE injury detection tool in female basketball and volleyball players [42]. A useful assumption for explaining such contrast would be that Walbright et al. considered only LE injuries, whereas in overhead sports, the trunk and upper extremities play an important role and may sustain as many injuries as or more injuries than the lower extremities. The FMS composite score is based on the component scores, which each test a unique movement pattern and a specific body location. Hence, a person may score less than 14 due to either poor mobility or poor stability at the shoulder but not sustain an LE injury. Therefore, the FMS composite score may fail to detect persons predisposed to injury when considering injuries that occur in only one part of the body.

The second category consists of studies refuting the predictive validity of the FMS, including Walbright et al. (2017), and Kodesh et al. (2015) [42, 46]. Sex characteristics influence movement parameters such as mobility, flexibility, stability and overall movement behavior. Given that these parameters are the absolute targets of the FMS, a strong possibility of an association between FMS scores and sex can be assumed. That the FMS composite score does not have the same meaning in male and female populations was also demonstrated through the studies of Gnacinski et al. (2015) and Moore et al. (2019) [36, 51]. As a result, having athletes screened based on their sex appears to be beneficial in identifying those who are prone to injury. As few studies have compared male and female populations, further studies of FMS measurement equivalence are obviously needed. Other systematic reviews and meta-analyses on mixed or male samples, however, revealed that the association between the FMS composite score and subsequent injury does not support the use of the FMS as an injury prediction tool [35, 55].

The third category contains the studies of Chorba et al. (2010), Knapik et al. (2015), Pfeifer et al. (2019), Clay et al. (2016) and Gonzalez et al. (2018), which partially support the validity of the FMS in the prediction of injuries, and the latter two studies were carried out among female coxswains and open-weight rowers [28, 31, 43, 44, 47]. Both studies showed that the FMS was unable to identify at-risk athletes but that it could predict LBP, indicating that chronic injuries might be predicted using the FMS. However, it is difficult to clearly discuss these studies as many limitations, such as non-generalizable results due to including one rowing team as the sample, missing data on the exposure time of the athletes, failure to control confounding variables, and the use of different definitions of LBP, were reported for these studies. In addition, Clay et al. included rowers who reported LBP within 6 weeks prior to the study, whereas Gonzalez excluded them, and this may have affected the results. More recently, Seidi et al. (2021) in a prospective study revealed that females who are predisposed to developing LBP in the future, have significantly lower FMS scores. They also highlighted that the FMS shows promise for predicting individuals who are prone to LBP development during prolonged standing [56]. Although the current literature addresses the association between the FMS composite score and LBP, further research is required to clarify the ability of the FMS to predict LBP, but with due attention paid to the limitations outlined above.

Chorba et al. (2010) as well as Landis et al. (2018) documented an association between previous injury and the FMS composite score among collegiate football, volleyball and basketball players, demonstrating that injury history should be considered a key risk factor for future injuries, and that injuries can be predicted by the FMS [31, 41]. In this line, Bahr (2016) stated that although screening tests usually measure modifiable risk factors, non-modifiable risk factors such as sex and history of injury can be used for better identification of at-risk individuals [52].

All in all, the available literature shows promise that the FMS could be a useful predictor tool in female populations if it: (a) reaches higher sensitivity through adopting the pre-identified cutoff points according to the population being assessed, (b) considers the non-modifiable but effective risk factors, (c) is applied in conjunction with other physical readiness screening measures. Another factor that might improve predictive ability of the FMS among females could be playing level or in general, level of physical readiness. In other words, the FMS may be a more practical screening tool for amateur athletes or those who participate in recreational activities than those who involved in higher levels of sport and physical activity. However, there is no evidence in the current literature to support this hypothesis, and it needs to be evaluated through further prospective studies.

## Conclusion

Having summarized the literature pertaining to the use of the FMS in females, it is concluded that although the current literature shows promise regarding the validity of the FMS to identify injury risk among active females, concerns about this issue cannot be fully addressed through this review due to the contradictory nature of the current literature. Therefore, a future study should include a meta-analysis of the literature to determine the validity of the FMS as an injury predictor tool. Additionally, further well-organized studies are highly recommended to address the gaps highlighted in this review. Meanwhile, it is clear that the sensitivity and specificity of the recommended FMS cutoff of ≤ 14 has considerably decreased over time and using higher cutoff values in addition to screening individuals based on their sex may help increase the FMS sensitivity. The FMS component scores, on the other hand, seem to be useful for injury risk identification when taking into consideration the characteristics of the population being assessed.

## Data Availability

Data sharing not applicable to this article as no datasets were generated or analyzed during the current study.
